# Integrating Machine Learning and Microwave-Assisted Green Extraction: Total Colorimetric Response Assay-Based Optimization of *Opuntia ficus-indica* Seed Residues

**DOI:** 10.3390/molecules31060998

**Published:** 2026-03-16

**Authors:** Souad Khaled, Amokrane Mahdeb, Farid Dahmoune, Meriem Amrane-Abider, Mohamed Hamimeche, Lydia Terki, Hamza Moussa, Hichem Tahraoui, Nabil Kadri, Hocine Remini, Mohammod Hafizur Rahman, Lotfi Khezami, Farid Fadhillah, Fekri Abdulraqeb Ahmed Ali, Amine Aymen Assadi, Jie Zhang, Abdeltif Amrane, Khodir Madani

**Affiliations:** 1 Centre de Recherche en Technologies Agro-Alimentaires, Route de Targa Ouzemmour, Campus Universitaire, Bejaia 06000, Algeria; souad.khaled@crtaa.dz (S.K.);; 2 Mountain Agro-Systems Division, Institut National de la Recherche Agronomique d’Algérie, INRAA, Oued-Ghir, Bejaia 06120, Algeria; amokrane.mahdeb@inraa.dz; 3 Département des Sciences Biologiques, Faculté des Sciences de la Nature et de la Vie et des Sciences de la Terre, Université de Bouira, Bouira 10000, Algeria; 4 Faculté des Sciences de la Nature et de la Vie, Université de Jijel, Jijel 18000, Algeria; mohamed.hamimeche@univ-jijel.dz; 5 Laboratoire de Zoologie Appliquée et d’Ecophysiologie Animale (LZA), Faculté des Sciences de la Nature et de la Vie, Université de Bejaia, Bejaia 06000, Algeria; 6 Laboratoire de Biomathématiques, Biophysique, Biochimie, et Scientométrie (L3BS), Faculté des Sciences de la Nature et de la Vie, Université de Bejaia, Bejaia 06000, Algeria; 7 Laboratory of Biomaterials and Transport Phenomena (LBMTP), University Yahia Fares, Médéa 26000, Algeria; hichem.tahraoui@univ-setif.dz; 8 College of Engineering, Imam Mohammad Ibn Saud Islamic University (IMSIU), Riyadh 11432, Saudi Arabiaffadhillah@imamu.edu.sa (F.F.); feali@imamu.edu.sa (F.A.A.A.); 9 College of Sciences, Imam Mohammad Ibn Saud Islamic University (IMSIU), Riyadh 11432, Saudi Arabia; lhmkhezami@imamu.edu.sa; 10 School of Engineering, Merz Court, Newcastle University, Newcastle upon Tyne NE1 7RU, UK; 11 Ecole Nationale Supérieure de Chimie de Rennes, CNRS, ISCR—UMR6226, University Rennes, 35000 Rennes, France

**Keywords:** *Opuntia ficus-indica* (L.) Mill, microwave-assisted extraction, optimization, AlCl_3_ complexation response, KNN, dragonfly algorithm, multi-objective optimization

## Abstract

The valorization of agro-industrial by-products is a sustainable approach to recovering high-value bioactive compounds. In this study, *Opuntia ficus-indica* (L.) Mill. seed press residues were investigated as a source of phenolic and flavonoid compounds using microwave-assisted extraction (MAE). A multi-step optimization strategy was implemented, combining preliminary single-factor experiments (OVAT), response surface methodology based on a Box–Behnken design (BBD), and machine learning modeling using K-nearest neighbors coupled with the dragonfly algorithm (KNN_DA), followed by desirability-based validation. The effects of ethanol concentration (50–100%), microwave power (400–800 W), extraction time (2–4 min), and liquid-to-solid ratio (30–50 mL/g) were evaluated on Folin–Ciocalteu reducing capacity (FCRC), AlCl_3_ complexation response, and antioxidant activity assessed by DPPH radical scavenging and reducing power assays. Optimal conditions were identified at 50% ethanol, 800 W microwave power, 4 min extraction time, and a liquid-to-solid ratio of 47.28 mL/g. Under these conditions, FCRC reached 376.85 ± 0.23 mg GAE/100 g DW and 49.16 ± 0.33 mg QE/100 g DW for AlCl_3_ complexation response, with prediction errors of 2.80% and 0.82%, respectively. The optimized extracts exhibited enhanced antioxidant activity. These findings confirm MAE as a rapid and environmentally friendly technique and highlight the predictive performance of the KNN_DA model for process optimization.

## 1. Introduction

The prickly pear, the edible fruit of the nopal cactus, belongs to the genus *Opuntia* within the family Cactaceae [1,2]. *Opuntia ficus-indica* (L.) Mill., originally native to the American continent, has been widely disseminated and is now cultivated in several regions worldwide, including Africa and Australia. The species was introduced into the Mediterranean basin during the 16th century [3,4]. Due to its specific agro-ecological adaptability, OFI has long been cultivated in semi-arid and arid regions. *Opuntia ficus-indica* (L.) Mill. typically grows in regions with hot summers and cool winters and can survive at high temperatures well above 35 °C. It tolerates low rainfall environments with annual precipitation ranging approximately from 250 to 800 mm [5,6]. Its unique ability to thrive under harsh environmental conditions and produce significant biomass makes it a valuable crop, especially in areas where access to other fresh vegetables is limited [7,8]. Its primary use remains the production of edible fruits, with Mexico contributing about 44% of global production (~428,300 t/year), alongside significant cultivation in Italy, South Africa, and other countries [9]. The plant is widely used to combat soil erosion in arid regions and serves as a forage alternative during drought periods in North Africa [10]. Its primary use remains the production of edible fruits [11]. However, the cladodes are also valued—both as a vegetable and as a raw material in various industries for the extraction of mucilage and pectin. Additionally, they have shown potential in the treatment of various types of wastewaters. While the primary economic use of OFI remains fruit production—consumed fresh or processed into juice or marmalade [12,13]—its processing generates seeds that constitute 20–40% of the fruit dry weight, depending on the cultivar [14]. These seeds are rich in unsaturated fatty acids, particularly polyunsaturated fatty acids (PUFAs), which have been linked to reduced risks of cardiovascular, inflammatory, and autoimmune diseases [15]. Furthermore, cactus-derived products contain phenolic compounds with potent antioxidant activity, capable of protecting proteins, DNA, and lipids from oxidative damage, and are associated with reduced incidence of oxidative stress-related conditions such as diabetes, cancer, cardiovascular, and neurodegenerative disorders [16].

Traditionally, phenolic and flavonoid compounds from OFI have been extracted using conventional solvent-based methods [17,18]. However, these conventional techniques often require prolonged extraction times, large volumes of organic solvents, and offer limited efficiency, raising environmental and operational concerns. In response, microwave-assisted extraction (MAE) has emerged as a greener alternative, reducing extraction time, energy consumption, and solvent use while improving reproducibility and ease of operation [19]. MAE represents a promising approach to efficiently recover bioactive compounds from OFI seed residues while aligning with the principles of green chemistry [19].

Process optimization is critical to maximizing yields of target compounds. Artificial intelligence (AI) techniques, including artificial neural networks, support vector machines, random forests, and K-Nearest Neighbors (KNNs), have demonstrated considerable potential for modeling and optimizing complex extraction processes [20,21,22]. KNN, in particular, offers simplicity and robustness for moderate-sized datasets, and its performance can be enhanced using optimization algorithms such as the dragonfly algorithm (DA) [23,24].

This study aimed to valorize *Opuntia ficus-indica* (L.) Mill. seed press residues by extracting phenolic and flavonoid compounds using MAE. Residues were defatted, sieved into a uniform powder, and subjected to preliminary one-factor-at-a-time experiments to identify the following key variables: solvent type, ethanol concentration, microwave power, irradiation time, and liquid/solid ratio. A Box–Behnken design (BBD) based on response surface methodology (RSM) was employed to model the effects on FCRC and AlCl_3_ complexation response, producing predictive polynomial equations. Complementing this, a supervised KNN model optimized with the dragonfly algorithm (KNN_DA) was developed to predict FCRC and AlCl_3_ complexation response from input parameters. Both BBD and KNN_DA models were validated against experimental data under optimal conditions. Additionally, a MATLAB R2022b based software integrating KNN_DA with multi-objective optimization (MODA) was developed to simulate, predict, and optimize extraction outcomes, representing a novel approach to valorize OFI seed residues efficiently and sustainably.

## 2. Results

### 2.1. Independent Variables Screening

Numerous factors can influence the studied system; therefore, identifying the most impactful variables is essential. In this study, the influence of ethanol concentration, irradiation time, microwave power, and liquid-to-solid ratio on FCRC and FCRC and AlCl_3_ complexation response was initially investigated using a one-variable-at-a-time (OVAT) approach.

#### 2.1.1. Solvent Types and Ethanol Concentration Effect

Statistical analysis showed that solvent type significantly influenced Folin–Ciocalteu reducing capacity (FCRC) and AlCl_3_ complexation response (*p* < 0.05, Table 1). Ethanol yielded the highest values, with 296.59 ± 2.32 mg GAE/100 g DW for FCRC and 30.37 ± 0.96 mg QE/100 g DW for AlCl_3_ complexation response. These results highlight ethanol as an effective solvent for phenolic extraction from press residues. Its superior performance, combined with low toxicity, environmental compatibility, and affordability, makes it a favorable choice for such applications. However, extraction efficiency is affected by the ethanol concentration in the solvent system [25]. Therefore, different aqueous ethanol concentrations were evaluated to identify the optimal ethanol–water ratio for maximizing phenolic compound extraction (Table 1). The data showed that FCRC and AlCl_3_ complexation response increased significantly as ethanol concentration rose from 30% to 50%. However, a further increase in ethanol concentration (from 70% to 100%) resulted in a marked decline in extraction efficiency. Similar trends have been reported in previous studies [26,27]. This decline at higher ethanol concentrations may be attributed to the reduced polarity of the solvent system, which limits the dissolution and diffusion of phenolic compounds. As solvent polarity decreases, molecular mobility is also reduced, thereby lowering the solubility and diffusion coefficients of phenolics into the solvent [28]. The role of ethanol concentration in microwave-assisted extraction is closely related not only to solvent polarity but also to dielectric properties that influence microwave absorption and heating efficiency. In microwave-assisted extraction, solvent mixtures with higher water content exhibit greater dielectric constants and loss factors, leading to a more efficient microwave energy absorption and improved heating of the plant matrix. Conversely, increasing ethanol concentration reduces solvent polarity and dielectric properties, which can decrease microwave absorption and consequently affect extraction efficiency [29].

Overall, the beneficial effects observed at intermediate ethanol concentrations can be attributed to an optimal balance between enhanced solubility of phenolic compounds and reduced thermal stress on the extraction system [30]. This combination likely limits the thermal degradation of heat-sensitive bioactive compounds. Based on these findings, an ethanol concentration range of 50% to 100% was selected for subsequent RSM trials. Additionally, 50% ethanol was fixed as the solvent for the following single-factor experiments evaluating the effects of microwave power and irradiation time.

#### 2.1.2. Microwave Power and Time Irradiation Effect

The results of MW pretreatment on FCRC and AlCl_3_ complexation response are presented in Table 1. Both responses increased significantly with microwave power increasing from 200 to 500 W, and the most significant decrease in FCRC and AlCl_3_ complexation response is found at 700 and 800 W.

As previously reported [31], microwave power leads to a rise in temperature due to its heating effect. This thermal increase likely enhances mass transfer, thereby improving phenolic compound recovery up to an optimal power density. Beyond this threshold, however, excessive heat may cause degradation of bioactive molecules [26]. Although increasing microwave power generally enhances extraction efficiency by promoting cell disruption and improving mass transfer, excessive microwave energy may cause localized overheating within the plant matrix. This can potentially lead to thermal degradation or oxidation of phenolic compounds, particularly under prolonged irradiation times. Therefore, an optimal microwave power level is required to balance extraction efficiency and compound stability [29].

Based on this, a power range of 400–800 W was chosen for the RSM experiments, while 500 W was applied during the final single-factor tests.

To examine the effect of irradiation time on the FCRC and AlCl_3_ complexation response, a separate experiment was carried out at various extraction time from 1 to 5 min. Regarding Table 1, augmentation of the extraction time from 1 to 3 min accompanied by improved FCRC and AlCl_3_ complexation response. However, these responses showed a significant decrease after 4 min. Prolonged microwave exposure without temperature regulation may lead to thermal degradation of phenolic compounds [32,33,34]. Additionally, shorter extraction times help minimize energy consumption. Therefore, a duration range of 2–4 min was used in the RSM study, with 2 min retained for subsequent single-factor experiments involving variations in the liquid-to-solid ratio.

#### 2.1.3. Liquid-to-Solid Ratio Effect

The FCRC and AlCl_3_ complexation response of press residue increased significantly from 193.97 to 379.00 mg GAE/100 g DW and from 33.57 to 59.38 mg QE/100 g DW respectively, with increasing liquid-to-solid ratio from 10 to 40 mL/g. An increased liquid-to-solid ratio can enhance solvent diffusion into plant cells and promote phenolic desorption, thereby improving extraction yield. However, as noted by Bhuyan, Van Vuong, Chalmers, van Altena, Bowyer and Scarlett [35], higher solid concentrations lead to denser suspensions, reducing solvent efficiency in solubilizing released compounds. Based on these considerations, a liquid-to-solid ratio range of 30–50 mL/g was chosen for the RSM experiments.

On the other hand, the extraction experiments were conducted using a single batch of *Opuntia ficus-indica* seed press residues. In practice, the chemical composition of plant materials may vary depending on cultivar, geographic origin, climatic conditions, harvest period, and processing methods. Such variability may influence extraction efficiency and the concentration of phenolic and flavonoid compounds. Therefore, further investigations involving multiple batches of raw material are needed to evaluate the robustness of the optimized conditions and the predictive performance of the developed models under variable conditions.

### 2.2. Modeling and Fitting the Models Using RSM

#### 2.2.1. Analyzing Relationships Between FCRC and AlCl_3_ Complexation Response

**Figure 1:**
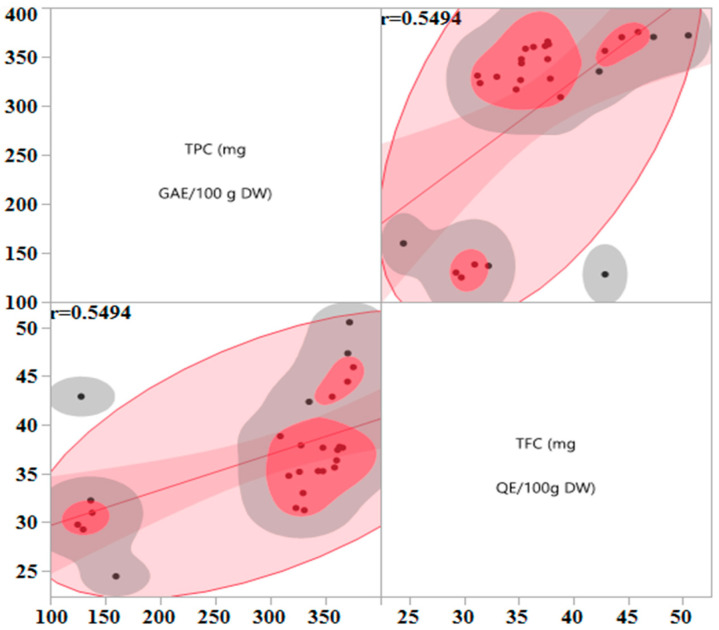
Relationship between FCRC and AlCl_3_ complexation response. Scatterplot showing the correlation between FCRC (mg GAE/100 g DW) and AlCl_3_ complexation response (mg QE/100 g DW) across different extraction conditions. Each point represents an experimental run.

This study employed a Box–Behnken design based on response surface methodology (RSM) with three central points to model the influence of four input variables—irradiation time, microwave power, ethanol concentration, and liquid-to-solid ratio—on FCRC and AlCl_3_ complexation response. To visualize the effects of these continuous variables and explore potential correlations between FCRC and AlCl_3_ complexation response, scatterplots were generated (Figure 1). According to Figure 1, the data points in the scatterplot for FCRC and AlCl_3_ complexation response are moderately clustered along the red line and a relationship appears to be between FCRC and AlCl_3_ complexation response (r = 0.5493); therefore, we can say that FCRC and AlCl_3_ complexation response might be influenced by input variables in a similar way.

#### 2.2.2. Experimental Design Analysis

Experiment trials of the press residue matrix were carried out according to the design matrix shown in Table 2. The goodness fit of the model of press residue FCRC and AlCl_3_ complexation response based on experimental data was analyzed by ANOVA. This includes tests of model significance test, their coefficients (intercept, linear, quadratic and interaction term) and lack of fit model adequacy (Table 3).

The fit summary for FCRC and AlCl_3_ complexation response suggested that both experiment models are significant, with *p* value < 0.01. The quality of the models was confirmed by like of R^2^ and adjusted R^2^ greater than 95%, while lack of fit *p*-values (0.12 for FCRC and 0.18 for AlCl_3_ complexation response) were not significant.

The difference between adjusted R^2^ and R^2^ is less than 0.2; in a good statistical model, adjusted R^2^ should be close to R^2^, which is a sign of good ANOVA model. Another test for assessing ANOVA adequacy is the root mean square error (RMSE), the square root of the variance of the residuals, whose values are 6.94 and 1.60 for FCRC and AlCl_3_ complexation response, respectively; a lower RMSE indicates a better fit and provides a good measure of how accurately the model predicts the response. Thus, the models obtained in the present work satisfy the ANOVA criteria and provide a good fit, which can be used for future predictions.

The input parameters, namely ethanol concentration (X_1_), microwave power (X_2_) irradiation time (X_3_) and liquid-to-solid ratio (X_4_) have highly significant influences on FCRC and AlCl_3_ complexation response as indicated by their less-than-0.01 associated *p*-value. Although the interaction parameters X_1_X_2_, X_2_X_3_ and X_3_X_4_ were not significant (*p* > 0.05) (in case of FCRC), X_1_X_3_ and X_1_X_4_ and quadratic parameters (X_1_^2^, X_3_^2^ and X_4_^2^) were significant (*p* < 0.05).

Regarding AlCl_3_ complexation response, the quadratic parameters were not significant (*p* > 0.05) except X_4_^2^ (*p* < 0.05). The interaction parameters X_1_X_2_, X_1_X_4_, X_2_X_3_ and X_3_X_4_ were also significant (*p* < 0.05).

The relationship between input and output variables is given in the following mathematical polynomial models (Equations (1) and (2)).

Y _FCRC_ = 362.78 − 109.01 X_1_ + 7.59 X_2_ + 12.62 X_3_ + 13.56 X_4_ − 10.38 X_1_X_3_ − 9.73 X_1_X_4_ − 105.74 X_1_^2^ − 13.87 X_3_^2^ − 15.98 X_4_^2^ (1)

Y_TFC_ = 37.23 − 5.59 X_1_ + 1.95 X_3_ + 1.56 X_4_ − 8.45 X_1_ X_2_ − 3.17 X_1_X_4_ + 2.97 X_2_X_3_+ 4.71 X_3_ X_4_ − 1.52 X_4_^2^ (2)

The experimental design data of press residue FCRC and AlCl_3_ complexation response recoveries were presented in Table 2 and Table 3. The FCRC and AlCl_3_ complexation response ranged from 125.02 to 375.26 mg GAE/100 g DW 24.42 to 50.53 mg QE/100 g DW, respectively. The polyphenol and flavonoid contents obtained in this study were substantially higher than those found in the OFI seed studied by [17,18]. These findings indicate that microwave-assisted extraction (MAE) is a promising alternative to conventional solvent methods for extracting phenolic compounds from *Opuntia ficus-indica* (L.) Mill. seed press residues, as it provides higher yields of bioactive compounds and enhanced antioxidant capacity.

#### 2.2.3. Analysis of the Contour Profiler Plots

**Figure 2:**
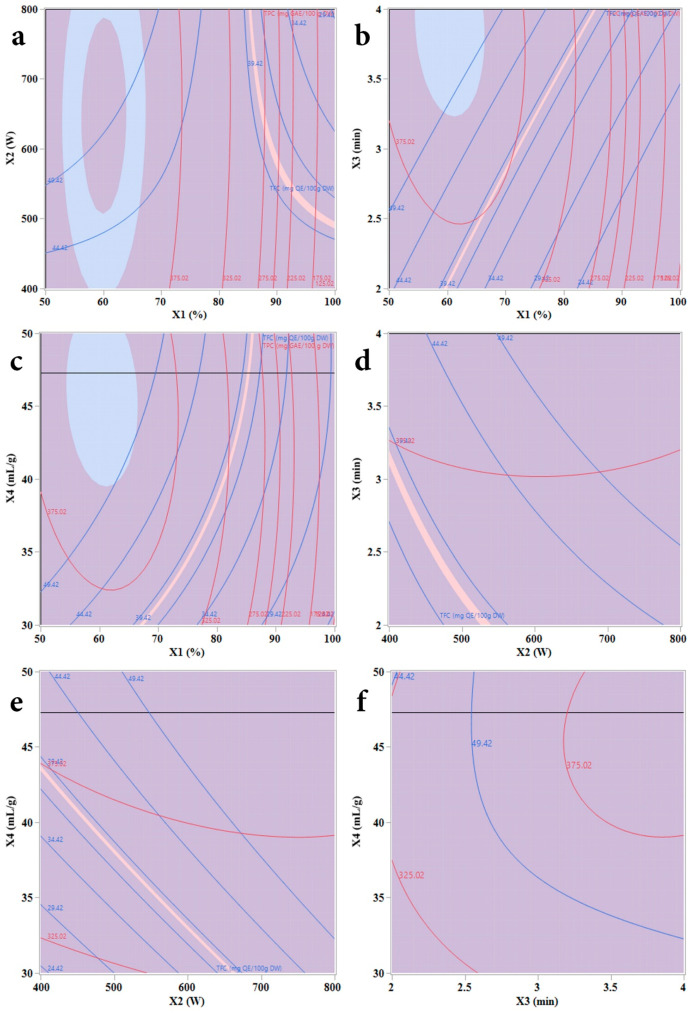
Contour plots of key extraction parameters affecting FCRC and AlCl_3_ complexation response yields. (**a**) Ethanol concentration vs. microwave power, (**b**) ethanol concentration vs. irradiation time, (**c**) ethanol concentration vs. liquid-to-solid ratio, and (**d**) microwave power vs. time; (**e**) microwave power vs. liquid-to-solid ratio, (**f**) time vs. liquid-to-solid ratio. The responses correspond to FCRC and AlCl_3_ complexation from *Opuntia ficus-indica* (L.) Mill. press residue seeds, represented by red and blue contour lines, respectively.

The combined effects of the independent variables crossing effects on the FCRC and AlCl_3_ complexation response of press residue can be seen on Figure 2. They show the type of interactions between two tested variables and the relationship between responses and experiment levels of each variable by maintaining the dependent variables at 396.97 to 403.25 mg GAE/100 g DW and 38.59 to 38.91 mg QE/100 g DW for FCRC and AlCl_3_ complexation response, respectively.

**Figure 3:**
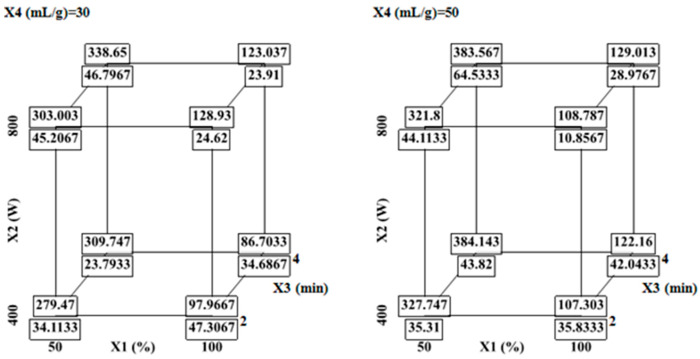
Cube plots of predicted FCRC and AlCl_3_ complexation response values at extreme factor combinations. Predicted FCRC (mg GAE/100 g DW) and AlCl_3_ complexation response (mg QE/100 g DW) for varying ethanol concentration, microwave power, extraction time, and liquid-to-solid ratio. Results are shown at two levels of liquid-to-solid ratio as follows: 30 mL/g and 50 mL/g.

It is observed that when all extraction parameters (ethanol, MW power, extraction time and liquid-to-solid ratio) are kept at their central level, the FCRC and AlCl_3_ complexation responses are 362.78 mg GAE/100 g DW and 37.38 mg QE/100 g DW, respectively. However, the cube plots, which display predicted values for the extremes of the factor ranges arranged at the vertices of cubes (Figure 3) show that there are two responses; therefore, FCRC and AlCl_3_ complexation response are shown stacked at each vertex. The FCRC and AlCl_3_ complexation response decreases to 279.46 mg GAE/100 g DW and 34.11 mg QE/100 g DW at low level (−1), and the decreasing reaches 129.01 mg GAE/100 g DW and 28.29 mg QE/100 g DW the bottom at high levels (+1) (Figure 3).

**Figure 4:**
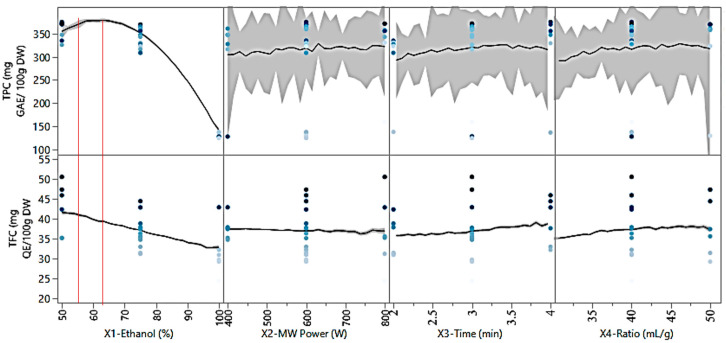
Prediction profiler showing the effect of extraction variables on FCRC and AlCl_3_ complexation response. The profiler illustrates the influence of ethanol concentration, microwave power, extraction time, and liquid-to-solid ratio on both responses. Black lines indicate model predictions; shaded regions represent confidence intervals. Dots represent experimental observations. The red vertical lines indicate the selected optimal levels of the extraction parameters used for the prediction of the responses.

The results show that the recoveries, as a function of ethanol concentration, are highly significant at the 95% confidence level due to the very low *p* value (<0.0001). A higher percentage of ethanol, above 64%, leads to the extraction process, resulting in less FCRC and AlCl_3_ complexation response (Figure 4).

The ethanol/water ratio played an important role in increasing and decreasing the bioactive yield. The optimum ethanol/water ratio was found to be around 60 to 64% and decreasing or increasing the solvent ratio (<60 or >64%) lowered the phenolic yield over the range of other parameters (Figure 4).

We can say that ethanol concentration was perhaps the most important factor that significantly influenced the FCRC and AlCl_3_ complexation response. Karazhiyan et al. [36] found that ethanol concentration plays a critical role in the extraction of bioactive compounds from various natural products. Similarly, these results agreed with our previous studies on phenolic compounds present in *Myrtus communis* leaves, in which we reported that solvent concentration was the most important factor contributing to the extraction of phenolic components using RSM.

The response surfaces were visualized in three dimensions by plotting the response variable on the *z*-axis against combinations of three independent variables, while maintaining ethanol concentration near its optimal value (~60%). Each 2D contour plot (Figure 2) illustrates the interaction between two variables across a range of values. These plots, derived from the regression model, offer a valuable visual tool for assessing the relationships between input factors and extraction yield. The shape of the contours provides insight into interaction effects: circular contours imply minimal interaction, whereas elliptical shapes indicate significant interactions between the paired variables.

The interaction effects of ethanol concentration with MW power and liquid-to-solid ratio contributed negatively to the response (Table 3). Figure 2 and Figure 3 show that the increasing MW density from 13.33 to 16 W/mL increases the responses from 337 to 387.57 mg GAE/100 g DW and 45.36 mg QE/100 g Dw respectively at 3 min of extraction time. The overall phenolic yield decreased significantly when the ethanol reached 100%. The reduced FCRC and AlCl_3_ complexation response at higher ethanol concentrations may be attributed to the decreased polarity of the solvent mixture, which limits molecular mobility. This reduction in solvent polarity likely lowers the diffusion coefficient and solubility of phenolic compounds, thereby hindering their effective extraction.

In relation to microwave heating and solvent polarity, it is noteworthy that water has a significantly higher dielectric constant than ethanol (ε′ = 80.4 vs. 24.3), but a lower dielectric loss factor (22.8 vs. 8.52 at 25 °C). This implies that water absorbs microwave energy more slowly but allows for deeper penetration of the radiation. Consequently, the beneficial effects observed with moderate ethanol concentrations may result from a balance between enhanced solubility of phenolic compounds and reduced thermal stress, thereby minimizing degradation of FCRC and AlCl_3_ complexation response during extraction. Additionally, due to the nature of the colorimetric assays used to quantify FCRC and AlCl_3_ complexation response, higher measured values following heating beyond 3 min could be explained by the disruption of plant cell walls, facilitating the release of bioactive compounds into the solvent matrix [37].

Likewise, Karazhiyan, Razavi and Phillips [38] observed for cress seed (*Lepidium sativum*) extract that phenolic recoveries extracted with 50% ethanol were higher than those obtained by 100% ethanol. Usually, the ethanol–water blend solvent depends on phenolic compounds of plant materials and their extraction follows the ‘‘like dissolves like’’ principle [39].

Although microwave-assisted extraction has shown high efficiency at the laboratory scale, its application at larger scales may present certain challenges. In particular, factors such as microwave penetration depth, temperature homogeneity, and energy distribution can affect extraction efficiency in larger volumes. In addition, the cost of industrial microwave equipment and the need for process optimization may represent constraints for large-scale implementation. Further studies at pilot and industrial scales are therefore necessary to assess the technical and economic feasibility of scaling up the optimized extraction process.

### 2.3. K-Nearest Neighbors Coupled with Dragonfly Algorithm

Unlike response surface methodology, which assumes a quadratic relationship between variables and responses, the KNN_DA model is a non-parametric approach capable of capturing complex nonlinear interactions between extraction parameters. This allows for improved prediction accuracy, particularly near optimal conditions [40].

As previously reported, the development of the KNN model involved the systematic optimization of eleven distinct distance measures, including Euclidean, Chebyshev, Minkowski, Mahalanobis, Cosine, Correlation, Spearman, Hamming, Jaccard, Cityblock, and Standardized Euclidean. Each of these metrics was evaluated in combination with the following three types of distance weighting methods: Equal, Inverse, and Squared Inverse. To further enhance model performance, the DA was applied to fine-tune key parameters specific to each metric, such as the number of nearest neighbors and, for Minkowski distance, the power parameter (exponent) (Table 4).

The outcomes of this comprehensive optimization are detailed in Table 4, which reports statistical indicators such as the R and RMSE for the training set, validation set, and the full dataset (training + validation). These results are presented according to the optimal values of the relevant hyperparameters. The table also includes the complete list of distance metrics with their associated weighting techniques, as well as the DA configuration parameters employed in the tuning process.

Table 4 shows the results of the best model obtained after optimizing the KNN model using the DA algorithm. The optimization parameters used were 100 maximum iterations and 30 search agents, allowing the algorithm to efficiently traverse the search space while balancing exploration and exploitation.

For FCRC prediction, the optimization showed that the Jaccard distance, with an inverse squared distance weight and a neighbor count of 2, gave the best results. The R for the training set was 0.99, for the validation set 0.99, and for the total set 0.99. These values indicate an excellent predictive ability across all three phases. The RMSE for the training set was 0.8966, for the validation set 0.6381, and for the total set 0.7521 (Table 4). These results confirm that the model generalizes well and provides consistent accuracy across data subsets.

**Figure 5:**
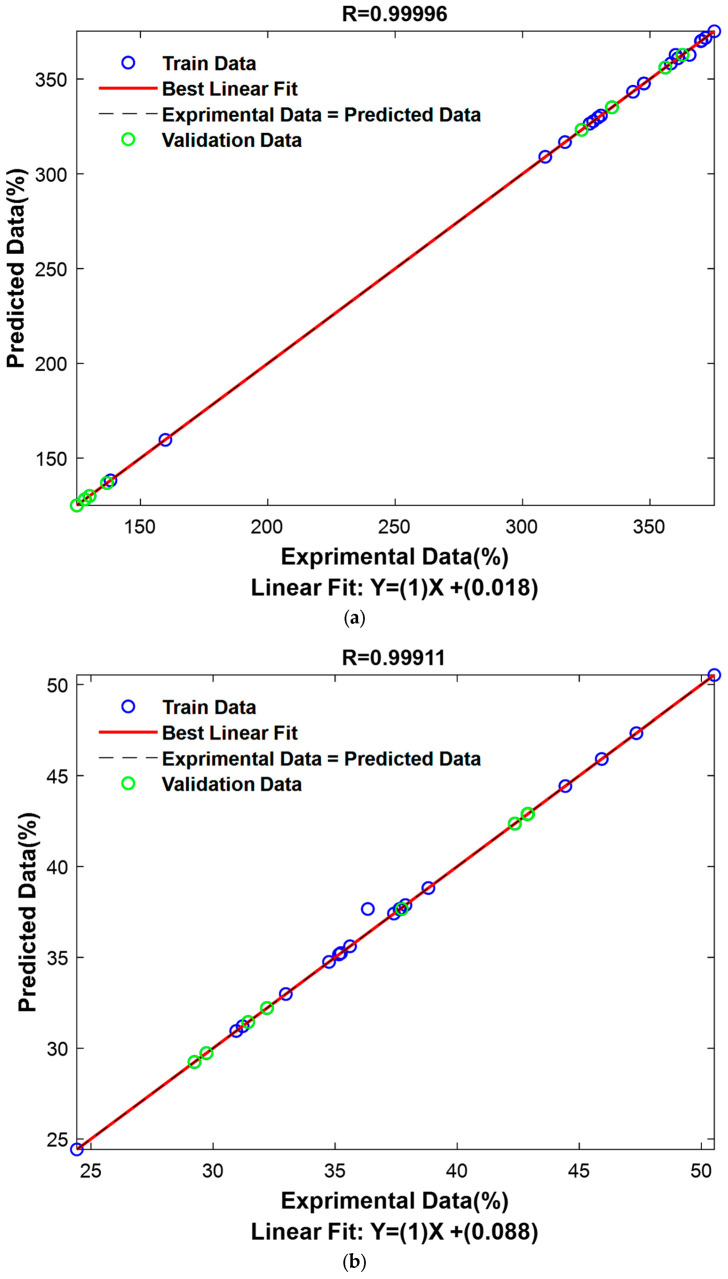
Correlation between experimental and KNN_DA-predicted values for FCRC and AlCl_3_ complexation response. (**a**) FCRC, mg GAE/100 g DW, and (**b**) AlCl_3_ complexation response, mg QE/100 g DW predicted by the K-nearest neighbor discriminant analysis (KNN_DA) model versus experimental values. The diagonal line represents the ideal prediction.

Regarding the AlCl_3_ complexation response prediction, the optimized model also used the Jaccard distance, an inverse squared distance weight, and a neighbor count of 1. The R for the training set was 0.9988, for the validation set 0.9999, and for the total set 0.9991, indicating near-perfect correlation between predicted and actual values, especially for the validation phase. The RMSE values were 0.3035 for the training set, 0.2381 for the validation set, and 0.2649 for the total set, further supporting the model’s high precision. The results for both models show exceptional performance, with R consistently above 0.99 and RMSE values remaining low, particularly for the AlCl_3_ complexation response validation set. These outcomes confirm the effectiveness of the DA algorithm in optimizing KNN model parameters. Moreover, the AlCl_3_ complexation response prediction model outperforms the FCRC model in terms of overall accuracy. Nevertheless, the FCRC model still demonstrates strong predictive ability and reliable generalization. These results are visually presented in Figure 5, providing a graphical representation of the findings.

### 2.4. Optimization and Validation of the Optimum Conditions

Multi-objective optimization based on the dragonfly algorithm (MODA) represents a bio-inspired strategy for optimizing bio-inspiration particulate effect to find complex problems in multiple objects. Inspired by the swarming behavior of dragonflies and their ability to alternate between exploration (global search) and exploitation (local search), the algorithm simulates social interactions (attraction, repulsion, and alignment) to guide a population of solutions along the Pareto optimal front. MODA allows you to explore more non-dominant solutions that offer a rich view and diversify possible compromising objects into conflicts (for example, it maximizes the effectiveness of the environment and minimizes the impact on the environment). It has a flexible structure, has dynamic adjustment capacity and is efficient in removing mini-mode locations from MODA and a participatory method to adapt to complex systems, non-linear and large dimensions. By providing a set of optimal solutions, the dragonfly algorithm enables robust multi-criteria optimization and facilitates adaptation to real-world conditions. To simultaneously enhance both FCRC and AlCl_3_ complexation response, the MODA Algorithm was applied to fine-tune independent variables. The optimal solutions generated by the algorithm were then tested experimentally using the BBD approach combined with the KNN_DA model. A detailed comparison of predicted and experimental outcomes, along with the corresponding deviations, is provided in Table 5. The performance of the optimization models was experimentally evaluated to verify the reliability of the predicted optimal conditions. Two approaches were compared: the BBD-based approach and the KNN_DA regression model-based approach.

For the optimal solution provided by BBD (X_1_ = 51.98, X_2_ = 800, X_3_ = 4, and X_4_ = 48.18), the experimental values obtained were 375.85 ± 0.46 mg GAE/100 g DW for FCRC and 49.16 ± 0.37 mg QE/100 g DW for AlCl_3_ complexation response. However, the BBD model’s predictions overestimate these values, with a predicted FCRC of 407.21, an AlCl_3_ complexation response of 57.58, and a total of 464.79. The differences between the experimental and predicted values are therefore significant, reaching 31.36 for the FCRC, 8.43 for the AlCl_3_ complexation response, and nearly 39.79 in total. This overestimation suggests that although the BBD model was able to capture the general trends in the data, it has some limitations in terms of predictive accuracy in this optimal range.

In contrast, the solution provided by the KNN_DA model (X_1_ = 50.00, X_2_ = 800, X_3_ = 4, and X_4_ = 47.28) shows a much better match between the experimental and predicted values. The experimental FCRC is 376.85 ± 0.23 mg GAE/100 g DW, very close to the predicted value of 379.65, with a deviation of only 2.80. Similarly, the measured AlCl_3_ complexation response is 49.16 ± 0.33 mg QE/100 g DW, compared to a prediction of 48.34, a minor error of 0.82. The combined total presents an overall deviation of 2.99, demonstrating excellent accuracy of the KNN_DA model.

These results confirm that, although both approaches can generate experimental conditions close to theoretical optima, the KNN_DA model stands out for its much better predictive ability. It significantly minimizes prediction errors compared to experimental data, making it the best validated model for simultaneously predicting maximum FCRC and AlCl_3_ complexation response contents under the experimental conditions tested.

### 2.5. Antioxidant Activity

To evaluate the antioxidant potential of extracts from OFI seeds, the following two complementary tests were carried out: the antiradical capacity test (DPPH) and the reducing power (RP) test. These analyses were carried out on extracts obtained under the optimal conditions determined by the KNN_DA model, which maximize the FCRC and AlCl_3_ complexation response. The results obtained are presented in Table 6.

The DPPH radical scavenging activity of *OFI* seed press cake extract reached 39.57% under the tested conditions, indicating a substantial antiradical capacity. Comparable results have been reported for *OFI* seed extracts, where radical scavenging activity was shown to increase in a concentration-dependent manner and, in some cases, exceed 50% at higher extract concentrations [41]. The evaluation of the reducing power of the extraction residue from *Opuntia ficus-indica* (OFI) seeds revealed a concentration of 14.35 mg AAE/100 g DW, reflecting a moderate electron-donating capacity of the antioxidant compounds present. This value is generally consistent with data reported in the literature, although substantial differences may be observed depending on extraction conditions, cultivars, and analytical methods employed. For instance, Chougui, Tamendjari, Hamidj, Hallal, Barras, Richard and Larbat [18] reported total phenolic contents in *O. ficus-indica* seeds ranging from approximately 32.3 to 51.3 mg AAE/100 g DW. Similarly, Chaalal, Louaileche, Touati, Bey and Products [17] reported higher reducing power values than those observed in the present study, ranging from approximately 18.61 to 19.78 mg AAE/100 g DW. More recently, Bouaouich, Bouguerche, Mahiaoui, Peron and Bendif [42] evaluated the reducing power of Algerian *O. ficus-indica* seeds and obtained values between 8.89 and 30.1 mg AAE/100 g DW, depending on the cultivar, which partially overlaps with our findings.

These antioxidant properties could be explained by the presence of bioactive compounds, particularly polyphenolic compounds. Indeed, Osuna-Martínez, Reyes-Esparza and Rodríguez-Fragoso [16] reported that these latter are the main phytochemicals responsible for the antioxidant activity of fruits and vegetables. These values confirm that the optimal extraction conditions enable the recovery of extracts rich in bioactive compounds with significant antioxidant potential. These performances are explained by the ability of phenolic compounds to neutralize free radicals and reduce oxidizing agents, thus helping to prevent oxidative damage to biological macromolecules. The integration of the KNN_DA model into the experimental optimization made it possible to identify an efficient extraction configuration, demonstrating the interest of coupling advanced analytical techniques with artificial intelligence tools for the valorization of agro-industrial residues.

The modeling approaches used in this study also present certain limitations. Response surface methodology is based on polynomial equations and may not fully describe complex nonlinear relationships outside the studied experimental domain. Similarly, the performance of machine learning models depends strongly on the size and quality of the dataset. With relatively small experimental datasets, there is a potential risk of reduced generalization capability. Therefore, the predictive models developed in this work should be interpreted within the limits of the experimental conditions and may benefit from further validation using additional experimental data.

### 2.6. Interface for Optimization and Prediction

**Figure 6:**
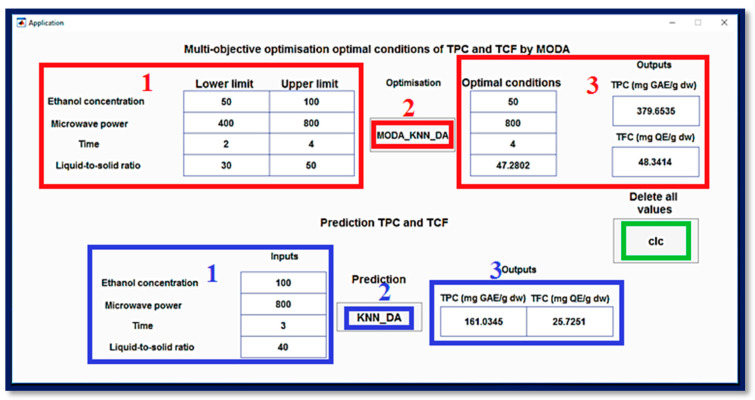
MATLAB interface for multi-objective optimization using MODA and KNN_DA prediction. The graphical interface illustrates the application of the multi-objective dragonfly algorithm (MODA) for simultaneous optimization of FCRC and AlCl_3_ complexation response, using predictions from the KNN_DA model. The red and blue numbers indicate the sequential steps for using the interface.

A software tool was developed in this work to forecast FCRC and AlCl_3_ complexation response based on predictive models generated by the KNN_DA algorithm, identified as the best-performing model in this study (Figure 6). The tool enables users to enter specific experimental parameters such as ethanol concentration (X_1_: 50–100%), microwave power level (X_2_: 400–800 W), extraction duration (X_3_: 2–4 min), and liquid-to-solid ratio (X_4_: 30–50 mL/g) to obtain corresponding predicted values of FCRC and AlCl_3_ complexation response. The application is also equipped with a multi-objective optimization function relying on the MODA Algorithm, which determines optimal conditions within the studied domain for the simultaneous enhancement of both FCRC and AlCl_3_ complexation response. By scanning the defined experimental domain, the algorithm outputs candidate optimal conditions according to the KNN_DA predictions. These optimal configurations are then tested in the laboratory to confirm their reliability. Through a graphical interface, the application provides output data, including predicted responses and optimal parameter settings, allowing users to assess the performance of the predictive models and observe how different factors influence the results.

However, the predictions generated by this tool remain limited to the experimental domain and to the OFI seed press residues investigated in this study. The extraction efficiency and optimal conditions are expected to vary depending on the physicochemical characteristics of different plant matrices. Therefore, although this modeling approach may be adapted to other plant materials, its application would require additional experimental validation to ensure reliable predictions.

## 3. Materials and Methods

### 3.1. Chemical Reagents

Folin–Ciocalteu reagent and aluminum chloride were obtained from Biochem Chemopharma (Montreal, QC, Canada), while sodium carbonate and sulfuric acid were sourced from Biochem Chemopharma (Savannah, GA, USA). Gallic acid was purchased from Biochem Chemopharma (London, UK). Organic solvents including acetone, ethanol, methanol, and butanol were supplied by Prolabo (Paris, France). All other reagents were procured from Sigma–Aldrich GmbH (Darmstadt, Germany).

### 3.2. Plant Material

The seed press residue of *Opuntia ficus-indica* (L.) Mill. was obtained after mechanical cold-press oil extraction from mature fruits collected in Bejaia, Algeria, during the 2023 harvesting season. The plant material was botanically identified at the Department of Biology, University of Bejaia, and processed from a single production batch to minimize variability.

The collected press residue was air-dried to constant weight and ground using an A11 basic analytical mill (IKA, Staufen, Germany). The resulting powder was sieved through a 500 µm stainless-steel mesh, and only particles smaller than 500 µm were retained to ensure homogeneity and improved extraction efficiency. The moisture content of the powdered material was determined prior to extraction. Defatting was performed using a Soxhlet apparatus (Behr Labor-Technik, Düsseldorf, Germany) with n-hexane as solvent for 6 h to remove residual lipids. After extraction, the defatted material was dried at room temperature under a fume hood to eliminate solvent traces. The final powder was stored in airtight glass containers at 4 °C in the dark until further analysis.

### 3.3. Microwave-Assisted Extraction

The phenolic compounds from *Opuntia ficus-indica* (L.) Mill. press residue powder were extracted using a domestic digital microwave oven (2450 MHz, 230 V, 50 Hz; Samsung Model NN-S674MF, Kuala Lumpur, Malaysia). This microwave system featured a digital control panel allowing adjustable power settings from 100 to 1000 W and programmable irradiation times. To prevent solvent loss and ensure controlled extraction, the microwave cavity was perforated at the top and connected to an external vapor condensation system. This modification enabled continuous condensation and reflux of solvent vapors back into the extraction vessel during irradiation [20].

Extraction was performed using different solvents (distilled water, ethanol, methanol, and acetone). For each experiment, 1 g of *Opuntia ficus-indica* press residue powder was mixed with the appropriate solvent volume according to the solid-to-liquid ratios defined in the experimental design (Table 1 and Table 2) in a 500 mL glass flask. The mixtures were then subjected to microwave irradiation at the specified power levels and extraction times.

Although the temperature was not directly monitored during irradiation, all experiments were conducted under identical operating conditions (microwave power, irradiation time, solvent volume, and sample mass) to maintain reproducibility and ensure comparability between runs. Immediately after irradiation, the extraction vessels were rapidly cooled in an ice-water bath maintained at approximately 4 °C to terminate the extraction process and minimize thermal degradation of heat-sensitive compounds.

The extracts were subsequently filtered through Whatman No. 1 filter paper and transferred into volumetric flasks. All extracts were stored at 4 °C in the dark until further analysis. Each extraction experiment was performed in triplicate to verify reproducibility.

### 3.4. Colorimetric Assays

#### 3.4.1. Folin–Ciocalteu Reducing Capacity (FCRC)

FCRC was measured using the Folin–Ciocalteu method [43], with slight modifications. In brief, 500 µL of extract was mixed with 2.5 mL of 10% Folin–Ciocalteu reagent, followed by 2 mL of 7.5% Na_2_CO_3_ after 2 min at 27 °C. The mixture was incubated at 50 °C in the dark for 15 min, and absorbance was recorded at 760 nm using SpectroScan 50 spectrophotometer (Cecil Instruments, Cambridge, UK). FCRC was calculated from a gallic acid standard curve and expressed as mg GAE/100 g dry weight.

#### 3.4.2. ACl_3_ Complexation Response

AlCl_3_ complexation response was assessed following the method of [41], with minor adjustments. Equal volumes of extract and 2% AlCl_3_ (in methanol) were combined and incubated in the dark at room temperature for 15 min. Absorbance was read at 430 nm using a SpectroScan 50 spectrophotometer (Cecil Instruments, Cambridge, UK). AlCl_3_ complexation response was determined from a quercetin calibration curve and expressed as mg QE/100 g dry weight.

### 3.5. Determination of Antioxidant Activity

#### 3.5.1. Antiradical Activity (Radical DPPH)

The antioxidant activity (AA) of the extracts was assessed using the DPPH (1,1-diphenyl-2-picrylhydrazyl) radical scavenging assay, following the method of [44]. Briefly, 200 µL of *Opuntia ficus-indica* (L.) Mill. seed extract was added to 1000 µL of methanolic DPPH solution (60 µM). The mixture was incubated in the dark at room temperature for 30 min. Methanol, replacing the extract, was used as the control. The DPPH radical scavenging activity was calculated as percentage inhibition using Equation (3):

(3) $$DPPH\;scavenging\;activity\left(\%\right)=\frac{A_{Control}-A_{Sample}}{A_{Control}}\times 100$$

where $A_{Control}\;$ represents the absorbance of the control reaction and $A_{Sample}\;$ represents the absorbance in the presence of the extract.

#### 3.5.2. Reducing Power Assay (RP)

Reducing power was evaluated following [45], with slight modifications. One milliliter of extract was combined with 2.5 mL of phosphate buffer (0.2 M, pH 6.6) and 2.5 mL of 1% potassium ferricyanide, then incubated at 50 °C for 20 min. Afterward, 2.5 mL of 10% TCA was added. The upper layer (2.5 mL) was mixed with an equal volume of distilled water and 0.5 mL of 0.1% ferric chloride. Absorbance was measured at 700 nm. The results are expressed as equivalent mg Ascorbic acid (AAE) per 100 g of dry weight.

### 3.6. Experimental Optimization

The optimization procedure was conducted in two sequential steps. First, preliminary single-factor experiments (OVAT) were performed to determine the appropriate experimental ranges for each variable. Subsequently, a multivariate optimization was carried out using the Box–Behnken design (BBD), in which all variables were varied simultaneously to evaluate both individual and interaction effects on the extraction responses.

#### 3.6.1. Preliminary Trials

To optimize the microwave-assisted extraction process, the effects of key parameters—ethanol concentration, microwave power, irradiation time, and liquid-to-solid ratio—were selected as independent variables. The FCRC and AlCl_3_ complexation response served as the output variables. These factors were initially investigated individually through preliminary screening experiments to narrow down the experimental range and reduce the total number of trials (Table 1). During the evaluation of each parameter, the remaining variables were kept constant. The constant values used during the preliminary experiments were as follows: irradiation time (120 s), liquid-to-solid ratio (20 mL/g), ethanol concentration (50%), and microwave power (500 W). To investigate the effects of solvent type and ethanol concentration on FCRC and AlCl_3_ complexation response, microwave power was initially set at 500 W. During ethanol concentration trials, microwave power, irradiation time, and liquid-to-solid ratio were kept at 500 W, 2 min, and 20 mL/g, respectively. When testing microwave power, ethanol concentration, liquid-to-solid ratio, and irradiation time were fixed at 50%, 20 mL/g, and 2 min. For irradiation time analysis, constant values included 500 W, 50% ethanol, and 20 mL/g. Lastly, to evaluate the liquid-to-solid ratio, conditions were maintained at 500 W, 3 min, and 50% ethanol.

#### 3.6.2. Box–Behnken Experimental Design and Statistical Analyses

The Box–Behnken design (BBD), developed by Box and Behnken, offers an efficient and cost-effective alternative to other first- and second-order experimental designs, such as full factorial and central composite designs. BBD is structured to cover a cube domain when involving at least three independent variables and extends to a hypercube for four or more variables. It presents several advantages, including sequential properties, reduced number of required experimental runs, and high efficiency in modeling quadratic response surfaces. Additional characteristics of BBD include the requirement for a number of experimental trials calculated by the expression N = 2k(k − 1) + C_0_, where k is the number of factors and C_0_ is the number of center points [46]. Each factor is evaluated at the following three levels: −1, 0, and +1 [31]. In the present study, a BBD was applied to maximize the recovery of FCRC and AlCl_3_ complexation response from press residues (Table 1). The factors screened from the results of single-factor experiments on the response variables were: X_1_ ethanol concentration (50–100%), X_2_ microwave power (400–800 W), X_3_ extraction time (2–4 min) and X4 liquid-to-solid ratio (30–50 mL/g) (Table 7). The data were analyzed by JMP^®^ Version 13.0 (SAS Institute Inc., Cary, NC, USA) with a standard least square procedure to obtain RSM model. The ANOVA F test was used to assess model adequacy and coefficient significance at *p*-value less than 0.05.

The output results were fitted to a second-order polynomial equation, according to the model in Equation (4).

(4) $$Y=\beta_{0}+\sum_{j=1}^{k}\beta_{j}X_{j}+\sum_{j=1}^{k}\beta_{jj}X_{j}^{2}+\sum_{i}\sum_{<j=2}^{k}\beta_{ij}X_{i}X_{j}+e_{i}$$

In this model, Y represents the response function (FCRC or AlCl_3_ complexation response); $\beta_{0}$ is the intercept (constant term); $\beta_{j}$, $\beta_{jj}$, and $\beta_{ij}$ are the coefficients of the linear, quadratic, and interaction terms, respectively; and $X_{i}$ and $X_{j}\;$denote the independent variables.

#### 3.6.3. K-Nearest Neighbors Coupled with Dragonfly Algorithm

KNN is a supervised learning algorithm commonly used in classification and regression. It is based on a simple but powerful idea: to predict the value or class of an unknown point, the algorithm identifies the K closest observations in the training set based on a given distance and then aggregates the results from these neighbors. No explicit model is built in advance; the prediction is made only during the testing phase, by directly comparing the new data to already known examples. Compared to other machine learning techniques, KNN has several advantages [32]. First, it is non-parametric, meaning it makes no prior assumptions about the data distribution [47]. This makes it particularly useful for dealing with non-linear relationships or complex structures in the data. Furthermore, its implementation is simple, without requiring an expensive training phase as is the case for neural networks or complex tree models. It can therefore be quickly applied to various types of problems with little tuning. KNN also offers good readability of the decision-making process. The “neighborhood” principle is intuitive, which facilitates the interpretation of results, unlike more opaque algorithms such as random forests or support vector machines.

In this study, KNN models were developed to predict FCRC and AlCl_3_ complexation response using experimental data generated from the Box–Behnken design (BBD). The dataset consisted of the following four independent variables: ethanol concentration (X_1_: 50–100%), microwave power (X_2_: 400–800 W), extraction time (X_3_: 2–4 min), and liquid-to-solid ratio (X_4_: 30–50 mL/g).

Because the dataset originated from a structured experimental design, its size was inherently limited. Therefore, the objective of the machine learning approach was not to build a generalized predictive model but rather to compare modeling strategies and enhance prediction accuracy within the studied experimental domain.

Before training the models, the entire dataset was preprocessed by normalization in the range [–1, +1] to ensure homogeneous scaling of the variables. Next, the data were split into two groups as follows: 70% of the data was dedicated to training, while the remaining 30% was allocated to the model validation phase. This split was applied consistently across all tested models to ensure fair comparison between approaches.

To improve the prediction performance, a KNN parameter optimization phase was performed. This process involved the evaluation of 11 different distance metrics (including Euclidean, Mahalanobis, Minkowski, Chebyshev, Cosine, Correlation, Spearman, Hamming, Jaccard, Cityblock, and Seuclidean), each tested under three weighting schemes (uniform, inverse, and inverse squared distance). In addition, for each combination, the optimal value of the number of neighbors (k) was determined. To automate this optimization, the KNN algorithm was combined with the DA Optimization Algorithm. Renowned for its effectiveness in complex optimization problems encountered in machine learning, DA has enabled the systematic exploration of different KNN configurations, resulting in a high-performance hybrid model named KNN_DA. The latter ensures optimal integration of input parameters with available data, strengthening the predictive capacity of the model for estimating FCRC and AlCl_3_ complexation response under given extraction conditions.

Because the experimental dataset was limited in size, particular attention was given to minimizing overfitting and ensuring a fair comparison between models. To achieve this, identical datasets were used for all modeling approaches, and consistent training/validation splits were applied throughout the analysis. The hyperparameters of the KNN model were optimized based on validation error in order to improve predictive performance while avoiding overfitting. In addition to statistical evaluation, model predictions were experimentally validated under the optimal extraction conditions. The optimal conditions predicted by both the BBD and KNN_DA models were tested in the laboratory, and the experimental results were compared with the predicted values to assess model reliability. This experimental validation step provided an additional level of verification beyond statistical fitting and confirmed the predictive capability of the models within the studied experimental domain.

### 3.7. Statistical Analyses

In this study, the robustness of the BBD experimental design was examined using analysis of variance (ANOVA). All experiments were performed in triplicate (*n* = 3), and the results are expressed as mean ± standard deviation. The model fit was verified through the coefficient of determination (R^2^), as well as its corrected version, to estimate the proportion of variation explained by the model. The evaluation of the overall significance of the model was carried out using the Fisher test (F) and the statistical significance values (P), which allow judging the validity of the equations obtained. The impact of each independent factor, as well as the interactions between parameters, was analyzed via the *p*-values associated with each term of the model. To visualize the influence of the variables on the measured responses, two- and three-dimensional graphical representations were generated. The accuracy of the models was quantified using the root mean square error (RMSE). In addition, the predictive performances of the different variants of the KNN_DA model were studied using statistical indicators, notably the correlation coefficient (R) and the RMSE, whose mathematical expressions are based on previously established methodological approaches.

## 4. Conclusions

This study demonstrates the potential of microwave-assisted extraction (MAE) for the valorization of *Opuntia ficus-indica* (L.) Mill. seed press residues, an abundant agro-industrial by-product. By combining response surface methodology with machine learning, particularly a KNN model optimized by the dragonfly algorithm, an efficient approach for modeling and optimizing the FCRC and AlCl_3_ complexation response was developed. Under optimal conditions (50% ethanol, 800 W microwave power, 4 min irradiation time, and a 47.28 mL/g solvent-to-solid ratio), the extraction yielded 376.85 ± 0.23 mg GAE/100 g DW of FCRC and 49.16 ± 0.33 mg QE/100 g DW of AlCl_3_ complexation response. The KNN_DA model showed better prediction accuracy than the Box–Behnken design, confirming the usefulness of machine learning for optimizing extraction processes.

Beyond these findings, the study highlights the relevance of integrating green extraction techniques with data-driven modeling approaches to enhance the recovery of bioactive compounds from plant residues. This strategy contributes to the sustainable valorization of agro-industrial by-products and supports the development of natural antioxidant sources for potential food and pharmaceutical applications.

The experiments were conducted using a single batch of plant material under controlled conditions to isolate the effects of process variables. While this approach is appropriate for methodological optimization, natural variability between plant batches may influence extraction performance. Therefore, the optimized conditions and predictive models should be considered valid only within the studied experimental domain.

Future work should focus on scaling up the MAE process, identifying and characterizing individual phenolic compounds, evaluating antioxidant activity using standardized methods, and assessing the economic and environmental feasibility of the extraction process. Additionally, exploring further machine learning models may further improve prediction performance and optimization capacity.

## Figures and Tables

**Table 1:** Single-factor experiments for MAE.

		Press Residue	
Parameter		FCRC (mg GAE/100 g DW)	AlCl_3_ Complexation Response (mg QE/100 g DW)
Solvent nature (50%)	Pure water	161.62 ± 0.88 ^a^	23.18 ± 0.51 ^b^
	Ethanol	296.59 ± 2.32 ^c^	30.37 ± 0.96 ^c^
	Acetone	214.45 ± 1.31 ^b^	20.21 ± 0.55 ^a^
	Methanol	220.51 ± 3.48 ^ab^	21.77 ± 0.60 ^ab^
Ethanol concentration (%)	30	257.92 ± 1.91 ^a^	24.60 ± 0.81 ^b^
	50	300.90 ± 0.76 ^b^	36.35 ± 0.42 ^a^
	70	303.42 ± 1.16 ^b^	22.29 ± 0.64 ^c^
	100	138.52 ± 2.09 ^c^	20.62 ± 0.67 ^dc^
Microwave power (W)	200	228.35 ± 1.16 ^a^	24.91 ± 0.87 ^b^
	400	265.76 ± 2.87 ^b^	27.07 ± 0.16 ^c^
	500	303.42 ± 2.44 ^c^	32.57 ± 0.63 ^d^
	700	280.16 ± 1.58 ^d^	23.18 ± 0.51 ^a^
	800	220.77 ± 1.58 ^e^	26.07 ± 0.48 ^bc^
Extraction time (min)	1 min	243.26 ± 2.01 ^a^	27.91 ± 0.40 ^a^
	1.5 min	265.50 ± 2.44 ^b^	27.54 ± 0.72 ^a^
	2 min	303.67 ± 2.32 ^c^	24.97 ± 0.51 ^b^
	3 min	344.11 ± 1.52 ^d^	37.08 ± 0.79 ^c^
	4 min	242.50 ± 1.31 ^a^	16.94 ± 0.55 ^d^
	5 min	240.99 ± 0.76 ^a^	15.00 ± 0.48 ^e^
Liquid to solid (mL/g)	10	193.97 ± 0.76 ^a^	33.57 ± 0.79 ^a^
	20	344.11 ± 1.52 ^b^	43.22 ± 0.66 ^b^
	30	358.27 ± 1.16 ^c^	54.97 ± 0.48 ^c^
	40	379.00 ± 1.31 ^d^	59.38 ± 0.80 ^d^
	50	267.53 ± 3.48 ^e^	41.54 ± 0.32 ^e^

Results are reported as means ± standard deviation (S.D). ^a–e^: different letters indicate statistically significant differences at *p* < 0.05 according to ANOVA and Tukey’s post hoc test.

**Table 2:** Box–Behnken design with the observed responses and predicted values for yield of FCRC and AlCl_3_ complexation response referred to dry weight (dw) of OFI seeds (press residue) using microwave-assisted extraction. GAE, gallic acid equivalents.

Run	Pattern *X*_1_ *X*_2_ *X*_3_ *X*_4_	FCRC (mg GAE/100 g DW)		AlCl_3_ Complexation Response (mg QE/100 g DW)	
		Observed	Prediction	Observed	Prediction
1	+0+0	136.87 ± 0.47	136.40	32.21 ± 0.99	33.77
2	+00−	125.02 ± 1.42	128.23	29.73 ± 3.18	31.92
3	00−+	323.18 ± 3.32	327.34	31.44 ± 0.23	31.16
4	+−00	128.18 ± 3.59	133.38	42.90 ± 1.38	40.95
5	0+−0	330.76 ± 3.59	337.34	31.21 ± 1.18	32.52
6	00−−	308.91 ± 2.17	313.28	38.82 ± 0.60	37.44
7	00++	370.00 ± 2.74	365.65	44.43 ± 5.80	44.47
8	0000	365.56 ± 1.91	362.78	37.64 ± 0.45	37.24
9	0−0−	316.65 ± 1.42	313.08	34.75 ± 0.23	34.23
10	0+0−	343.35 ± 3.01	343.01	35.24 ± 1.38	34.39
11	0−0+	360.99 ± 0.72	354.95	37.41 ± 2.19	38.51
12	++00	159.78 ± 1.19	152.29	24.42 ± 0.60	23.07
13	+0−0	138.24 ± 3.98	131.92	30.95 ± 1.64	31.01
14	0−+0	347.62 ± 0.72	347.40	37.64 ± 0.60	37.41
15	−+00	371.79 ± 0.99	366.60	50.53 ± 1.77	51.15
16	−00+	370.21 ± 2.43	373.37	47.34 ± 3.16	46.23
17	0++0	356.04 ± 1.66	365.28	42.88 ± 1.18	42.38
18	−0+0	375.26 ± 1.71	375.20	45.92 ± 5.41	46.09
19	−−00	347.62 ± 3.68	355.12	35.23 ± 2.19	35.24
20	0000	362.73 ± 0.27	362.78	37.73 ± 1.59	37.24
21	0+0+	358.21 ± 4.67	355.39	35.61 ± 2.75	36.38
22	00+−	329.61 ± 3.28	322.46	32.98 ± 0.68	31.93
23	0000	360.05 ± 3.32	362.78	36.34 ± 3.87	37.24
24	−0−0	335.09 ± 2.24	329.17	42.36 ± 1.20	41.05
25	−00−	326.29 ± 1.19	326.78	35.16 ± 0.39	36.77
26	0−−0	327.71 ± 2.61	324.84	37.88 ± 1.27	39.47
27	+00+	130.00 ± 4.57	135.88	29.24 ± 0.68	28.72

**Table 3:** Analysis of variance (ANOVA) for the fitted quadratic polynomial model for optimization of extraction parameters.

Coefficient	Press Residue			
	FCRC		AlCl_3_ Complexation Response	
	Estimate	Prob > [t]	Estimate	Prob > [t]
β_0_	362.78	<0.0001 *	37.24	<0.0001 *
β_1_	−109.01	<0.0001 *	−5.59	<0.0001 *
β_2_	7.59	0.0026 *	−0.23	0.6484
β_3_	12.63	<0.0001 *	1.69	0.0055 *
β_4_	13.56	<0.0001 *	1.57	0.0050 *
β_12_	1.86	0.6027	−8.45	<0.0001 *
β_13_	−10.39	0.0113 *	−0.57	0.4758
β_14_	−9.74	0.0159 *	−3.17	0.0018 *
β_23_	1.342	0.7059	2.19	0.0472 *
β_24_	−7.37	0.0552	−0.57	0.4763
β_34_	6.53	0.0844	4.71	<0.0001 *
β_11_	−105.74	<0.0001 *	0.07	0.9187
β_22_	−5.19	0.1097	0.43	0.5533
β_33_	−13.87	0.0006 *	0.81	0.2752
β_44_	−15.98	0.0002 *	−1.66	0.0328 *
*P* of model >F		<0.0001 *		<0.0001 *
Lack of fit		0.1246		0.1826
R^2^	0.99		0.96	
R^2^Adj	0.99		0.93	

* Significantly different at *p* < 0.05; β_0_: intercept; β_1_, β_2_, β_3,_ and β_4_: linear regression coefficients for ethanol concentration, microwave power, irradiation time and liquid-to-solid ratio; β_12_, β_13_, β_14_, β_23_, β_24_, and β_34_: regression coefficients for interaction between ethanol concentration × microwave power, ethanol concentration × irradiation time, ethanol concentration × liquid-to-solid ratio, microwave power × irradiation time, microwave power × liquid-to-solid ratio and irradiation time × liquid-to-solid ratio; β_11_, β_22_, β_33_ and β_44_: quadratic regression coefficients for ethanol concentration × ethanol concentration, microwave power × microwave power, irradiation time × irradiation time and liquid-to-solid ratio × liquid-to-solid ratio.

**Table 4:** Summary of statistical indicators evaluating the predictive accuracy of the KNN_DA model for FCRC and AlCl_3_ complexation response, including R^2^, RMSE values.

DA: Max_iteration = 100, SearchAgents_no = 30								
Distance	Distance Weight	Num Neighbors	R			RMSE		
			Train	VAL	ALL	Train	VAL	ALL
FCRC								
Jaccard	Squared Inverse	2	0.9999	0.9999	0.9999	0.8966	0.6381	0.7521
AlCl_3_ complexation response								
Jaccard	Squared Inverse	1	0.9988	0.9999	0.9991	0.3035	0.2381	0.2649

**Table 5:** Comparison between actual yields and predicted responses from BBD and KNN_DA model for FCRC and AlCl_3_ complexation response under optimized extraction conditions.

BBD			
X_1_ = 51.9800, X_2_ = 800.0000, X_3_ = 4.0000, and X_4_ = 48.1750			
	FCRC	AlCl_3_ complexation response	FCRC + AlCl_3_ complexation response
FCRC experimental values	375.8514 ± 0.46	49.1563 ± 0.37	425,0077 ± 0.83
FCRC predicted values	407.2125	57.5826	464.7951
Error	31.3611 ± 0.46	8.4263 ± 0.37	39.7874 ± 0.83
**KNN_DA**			
**X_1_ = 50.0000, X_2_ = 804, and X_4_ = 47.2802**			
	FCRC	AlCl_3_ complexation response	FCRC + AlCl_3_ complexation response
FCRC experimental values	376.8514 ± 0.23	49.1614 ± 0.33	425,0077 ± 0.60
FCRC predicted values	379.6535	48.3414	427.9949
Error	2.8021 ± 0.23	0.82 ± 0.33	2.9872 ± 0.60

**Table 6:** DPPH• radical scavenging activity and reducing power of the extract obtained under optimal conditions predicted by the KNN_DA model.

Antioxidant Activities	
DPPH• Assay (%)	Reducing Power (mg AAE/100 g DW)
39.57%	14.35 ± 0.24

**Table 7:** Range of coded and actual values for Box–Behnken design.

Factor	Level		
	−1	0	+1
X_1_	50	75	100
X_2_	400	600	800
X_3_	2	3	4
X_4_	30	40	50

X_1_, ethanol concentration (% v/v, solvent/water); X_2_, power (Watt); X_3_, time (min); X_4_, ratio (mL/g).

## Data Availability

It will be available after acceptance and publishing in this journal.

## Abbreviations

The following abbreviations are used in this manuscript:

MAE	Microwave-assisted extraction
BBD	Box–Behnken design
KNN_DA	K-nearest neighbors coupled with dragonfly algorithm
ANOVA	Analysis of variance
RMSE	Root mean square error
FCRC	Folin–Ciocalteu reducing capacity
